# Bone Metabolism and RANKL/OPG Ratio in Rheumatoid Arthritis Women Treated with TNF-α Inhibitors

**DOI:** 10.3390/jcm10132905

**Published:** 2021-06-29

**Authors:** Agnieszka Jura-Półtorak, Anna Szeremeta, Krystyna Olczyk, Aleksandra Zoń-Giebel, Katarzyna Komosińska-Vassev

**Affiliations:** 1 Department of Clinical Chemistry and Laboratory Diagnostics, Faculty of Pharmaceutical Sciences in Sosnowiec, Medical University of Silesia, Katowice, 41-200 Sosnowiec, Poland; aszeremeta@sum.edu.pl (A.S.); olczyk@sum.edu.pl (K.O.); kvassev@sum.edu.pl (K.K.-V.); 2 Silesian Center of Rheumatology, Rehabilitation and Prevention of Disability of Gen. Jerzy Ziętek in Ustroń, 43-450 Ustroń, Poland; azongiebel@gmail.com

**Keywords:** rheumatoid arthritis, TNF-α inhibitors, bone turnover markers, PINP, PICP, NTX-I, CTX-I, RANKL/OPG

## Abstract

The aim of this study was to evaluate the effect of anti-tumor necrosis factor α (anti-TNF-α) therapy in combination with methotrexate on bone remodeling and osteoclastogenesis in female patients with RA. Serum levels of bone turnover markers (i.e., C- and N-terminal propeptides of type I procollagen (PICP and PINP), C- and N-terminal cross-linking telopeptides of type I collagen (CTX-I and NTX-I), and soluble receptor activator of nuclear factor κB ligand (sRANKL) and osteoprotegerin (OPG)) were determined by immunoassay at baseline and 15 months after initiation of treatment. Bone mineral density was measured by dual-energy x-ray absorptiometry. We found a significant decrease in serum PINP levels, a biomarker of bone formation, and higher levels of CTX-I and sRANKL indicative of increased bone resorption in RA patients prior to TNFαI treatment compared to the controls. Anti-TNF-α therapy was effective in improving bone metabolism in RA patients as reflected in a decrease in CTX-I (at least partially due to the RANKL/OPG reduction) and a concomitant increase in PINP levels. The bone metabolism changes were independent of the type of TNFαI used. PINP and CTX-I were found to be useful markers of bone metabolism, which may prove the effectiveness of TNF-α therapy earlier than the bone density assessment.

## 1. Introduction

Rheumatoid arthritis (RA) is a chronic systemic autoimmune disease of connective tissue characterized by symmetrical synovitis, eventually leading to bone erosion and cartilage damage. It affects approximately 0.5–1.5% of the world’s population and is about 2–3 times more common in women than in men, with an increased incidence in the age of 40–50 years. In the course of the disease, there are numerous extra-articular manifestations of the organs, which are the major causes of rapidly progressing disability, reduced quality of life, and increased mortality [1,2,3]. RA patients have more skeletal damage and a higher risk of fractures compared to the general population. The bone complications of RA include focal erosion of the marginal and subchondral bone, periarticular osteopenia, and generalized osteoporosis with reduced bone mass. High disease activity (persistent inflammation), long-term glucocorticoid therapy (>3 months), and the physical disability and immobility are the main factors that increase the risk of bone loss in patients with RA [3,4,5].

Bone is a highly dynamic tissue, undergoing continuous remodeling to maintain a healthy skeleton. Normal bone remodeling is an ongoing process in which osteoclast-mediated bone resorption is tightly coupled both temporally and spatially with osteoblast-mediated bone formation [6,7]. The coupling of these processes becomes disrupted in RA, resulting in the rapid breakdown of mineralized matrix and deterioration of bone microarchitecture [5,8,9,10].

Although the cellular mechanism of bone and cartilage destruction in RA is still not fully understood, both experimental and clinical findings indicate that pro-inflammatory mediators including tumor necrosis factor α (TNF-α) play a key role. TNF-α promotes osteoclastogenesis by inducing the expression of the essential osteoclast differentiation factor, the receptor activator of nuclear factor κB (NF-κB) ligand (RANKL), and/or its soluble receptor, osteoprotegerin (OPG), by bone marrow stromal cells of the osteoblast lineage or directly by enhancing the activity of cells in the osteoclast lineage [8,9,10,11,12,13,14].

The interaction between RANKL and its receptor-activator of nuclear factor κB (RANK) promotes the differentiation, maturation, activation, and survival of osteoclasts, leading to enhanced bone resorption and bone loss. RANKL/RANK signaling is controlled by a decoy receptor called osteoprotegerin, which competes with RANK for RANKL binding. The biological effects of OPG are opposite to those mediated by RANKL and include inhibition of end stages of osteoclast differentiation, activation of matrix osteoclast suppression, and accelerating osteoclast apoptosis [10,12,14,15]. Overall, the RANKL/OPG ratio determines the physiological balance of bone formation and turnover, with a higher ratio promoting increased bone resorption. It was found that the high RANKL/OPG ratio is associated with increased radiographic damage in RA patients [16]. Thus, the signaling and regulation of RANKL and OPG activity may play a critical role in bone loss associated with RA.

Anti-TNF-α therapies are effective in reducing inflammation and the progression of radiologic damage in RA patients and in murine models of arthritis [17,18,19], however, the mechanism by which TNF-α inhibitors (TNFαI) prevent the progression of bone destruction is still unclear. Therefore, the aim of this study was to investigate the effect of 15-month anti-TNF-α therapy on bone turnover markers and RANKL/RANK/OPG pathway in women with RA. We assessed the serum biomarkers indicative of bone remodeling—C-terminal propeptides of type I procollagen (PICP) and N-terminal propeptides of type I procollagen (PINP), which are markers of bone formation, and C-terminal crosslinking telopeptides of type I collagen (CTX-I) and N-terminal cross-linking telopeptides of type I collagen (NTX-I), which are markers of bone resorption—in female RA patients treated with TNFαI. However, according to the recommendation of the International Osteoporosis Foundation (IOF)–International Federation of Clinical Chemistry and Laboratory Medicine (IFCC) Working Group for Standardization of Bone Marker Assay (WG-BMA), only s-PINP and s-CTX-I are the reference bone turnover markers [20]. 

The results obtained in our research will supplement the knowledge of molecular mechanisms of action of selected TNF-α inhibitors used in the treatment of RA. Most of the studies conducted thus far have mainly assessed the effect of infliximab on bone remodeling in RA [21,22,23]. Only a few studies have included the assessment of the impact of other TNFα inhibitors analyzed in this study including etanercept, adalimumab, certolizumab pegol and golimumab on bone turnover biomarkers and osteoclast regulatory proteins [17,24,25]. Moreover, they included a short follow-up time, less than our number of patients as well as the absence of a control group. Thus, the aim of this study was to evaluate the usefulness of the quantitative serum assessment of individual markers of bone turnover as indicators of the clinical efficacy of anti-TNF-α therapy with etanercept and adalimumab in women with rheumatoid arthritis.

## 2. Materials and Methods

### 2.1. Patients and Samples

Fifty female patients that fulfilled the 1987 or 2010 American College of Rheumatology (ACR)/European League Against Rheumatism (EULAR) classification criteria for RA [26,27] were enrolled in the study. Baseline patient characteristics are presented in Table 1. All subjects had active RA with a 28 joint count disease activity score (DAS28) > 5.1 at baseline, despite taking at least two synthetic disease-modifying antirheumatic drugs (DMARDs). Exclusion criteria included prior treatment with biologic agents, acute or recent infection, concomitant diseases affecting bone metabolism, fractures, renal or liver insufficiency, heart failure, chronic alcoholism, pregnancy and breastfeeding. None of the enrolled subjects received bisphosphonates or hormone replacement therapy, which could have interfered with bone metabolism. In addition, none of the patients had smoked cigarettes for at least three months prior to the study. All participants received anti-TNF-α treatment combined with methotrexate (MTX) (25 mg/week) over a 15-month period. Biological agents were administered at recommended doses indicated in RA—for adalimumab (ADA; Humira) 40 mg every other week as subcutaneous (SC) injection, for etanercept (ETA; Enbrel) 50 mg once weekly as SC injection, for certolizumab pegol (CZP; Cimzia) 400 mg at 0, 2, 4 weeks, and then 200 mg every two weeks as SC injection and for golimumab (GLM; Simponi) 50 mg once a month as SC injection. Patients were also given prednisone in a dose of ≤7.5 mg/day and folic acid in the dose of 5 mg/day. The use of calcium (1 g/day) and vitamin D (800–1000 IU/day) supplements were permitted. Concomitant medications remained unchanged for the duration of the study.

Presented in Table 1 are the variables of the demographic and clinical data in rheumatoid arthritis patients who qualified for the treatment with TNF-α inhibitors that were obtained in our earlier investigations [28].

As controls, reference values of clinical and bone turnover parameters were obtained from 26 age- and gender-matched healthy volunteers from the Medical University of Silesia in Katowice, Poland. Subjects were selected after obtaining their medical history, clinical examination, and laboratory screening. Women with any medical conditions that interfere with bone metabolism or with surgery in the past three years were excluded. Moreover, all participants included in the study showed normal morphology and biochemical analysis. Demographic, clinical, and laboratory data of control subjects in the study is presented in Table 2.

None of the volunteers took glucocorticoids or any other pharmaceutics known to affect bone metabolism or smoked cigarettes. We chose women who were able to maintain a healthy weight and had a body mass index (BMI) < 25 kg/m^2^. We excluded pregnant women and those who had a bone fracture (within 12 months) or had been operated on in the last three months as well as women with any disease that disturbed bone metabolism.

This study was approved by the Ethical Committee of the Medical University of Silesia in Katowice (KNW/0022/KB/56/I/12/13). All participants gave written informed consent prior to the study, and research was carried out in accordance with the conditions of the Declaration of Helsinki.

### 2.2. Laboratory Parameters and Evaluation of RA Activity

Measurement of rheumatoid factor (RF; normal value ≤ 15 U/mL) and C reactive protein (CRP; normal value < 5 mg/L) was performed at Konelab Prime 30ISE, bioMérieux, France. The anti-CCP antibodies (normal value ≤ 5 U/mL) were determined by enzyme-linked immunosorbent assay (ELISA) from Euroimmun (Lubeck, Germany). In addition, erythrocyte sedimentation rate (normal range for women: 3–12 mm/h) was determined by the Westergren method (Sediplus^®^ S2000, Sarstedt, Germany). RA activity and response to TNFαI includes three variables with 28 joint counts of tenderness and swelling, and erythrocyte sedimentation rate (ESR) and the patient’s assessment of disease activity (three variables). Patients who did not respond well to treatment were excluded from the study. A good response was defined—in accordance with the assumptions of the Polish National Health Fund’s Therapeutic Programs—as a reduction in DAS28-ESR by more than 1.2 after the first three months of biological therapy and a further reduction in DAS28-ESR by 1.2 was noted in subsequent medical examinations performed at nine and 15 months after the first dose of TNFαI. 

### 2.3. Measurement of Bone Turnover Markers

Venous blood samples were collected between 7:00 am and 9:00 am after an overnight fast. Serum obtained from healthy subjects and RA patients was separated from whole blood after complete clotting by centrifugation at 3000 rpm for 10 min and immediately stored at −80 °C until analysis. All bone turnover markers were measured at baseline and 15 months after starting TNFαI treatment. Bone formation was analyzed on the basis of serum levels of PICP and PINP using an ELISA method from Cloud-Clone Corp. (Katy, TX, USA). The minimum detectable concentration was <0.067 ng/mL for PICP and <13.2 pg/mL for PINP. Serum levels of bone resorption markers, (i.e., CTX-I and NTX-I) were determined using a competitive enzyme immunoassay from Immunodiagnostic Systems Ltd. (Boldon, UK) and competitive inhibition ELISA using the Osteomark^®^ assay (Alere Scarborough, Inc., Scarborough, ME, USA), respectively. Detection limit for CTX-I was 0.020 ng/mL. The NTX-I serum assay values were expressed in nanomoles bone collagen equivalents per liter (nM BCE). The reference range was between 3.2 and 40.0 nM BCE. The manufacturer’s recommended reference values for women ranged from 6.2 to 19 nM BCE. The levels of the markers of osteoclastogenesis, (i.e., total soluble RANKL and OPG) were measured using commercially available ELISA kits from BioVendor R&D (Brno, Czech Republic) according to the manufacturer’s instructions. The minimum detectable concentration was estimated to be 0.40 pmol/L for sRANKL and 0.03 pmol/L for OPG. Testing of all samples in duplicate was completed in one day to eliminate the effects of inter-assay variation. The manufacturer’s intra-assay coefficients of variation (CVs) were <10% for PICP, PINP and sRANKL, <3% for CTX-I, = 4.6% for NTX-I, and <4.9 for OPG. 

### 2.4. Measurement of Bone Mineral Density

Bone mineral density (BMD) was measured in women with RA at baseline and 15 months after the first TNFαI course by dual-energy x-ray absorptiometry (DXA). DXA examination was performed using the Hologic Horizon Wi (Hologic Inc., Waltham, MA, USA) densitometer by a single technician during the study period. Measurements were taken at the posterior–anterior lumbar spine (region L2–L4) and left proximal neck of femur (femoral neck). In our study, the BMD (g/cm^2^), the rate of T-score (density of bone in comparison with young people), and the rate of Z-score (density of bone in comparison with their peers) were studied. The inter-assay coefficient of variation (CV), measured using an anatomical spine phantom daily was less than 1.8.

### 2.5. Statistical Analysis

Data analyses were performed using TIBCO Software, Inc. (1984–2017); StatSoft Poland Sp. z o. o. 2021 (Palo Alto, CA, USA). The normality of the distribution was verified using the Shapiro–Wilk test. Data not normally distributed were log-transformed before the analyses. Variables are summarized as mean ± SD (for normal distribution) or median and interquartile (25th–75th percentile) range (for abnormal distribution). The homogeneity of variance was assessed by Levene’s test. Evaluation of data was carried out using a repeated measures analysis of variance (RM-ANOVA) (normal data distribution) with a sphericity check employing Mauchly’s test of sphericity, or using the RM-ANOVA Friedman’s test (non-normal data). Post-hoc analyses performed in cases of significant differences between subgroups relied on the Tukey’s test (*p* value < 0.05) or the Mann–Whitney U-test (*p* value obtained after application of the Bonferroni correction, *p* < 0.05/six possible comparisons). Moreover, the Mann–Whitney U-test was used to determine whether the differences between the values for RA patients, both at the beginning and after 15 months of TNFαI therapy, were significantly different from the control group. Paired Student’s t-test (for normal distribution) or Wilcoxon’s rank sum test (for abnormal distribution) was used to compare the change in the same parameters in each RA patient before and after 15 months of anti-TNF-α treatment. *p* values of less than 0.05 were considered significant. Spearman’s rank correlation coefficient was used to evaluate the relationship between selected biomarkers of both bone turnover (PINP, CTX-I and PINP/CTX-I ratio) and osteoclastogenesis (sRANKL, OPG, sRANKL/OPG ratio) in women with RA. The significance in case of multiple comparisons was assessed against a reference *p* value obtained after applying the Bonferroni correction (*p* < 0.05/six possible comparisons).

## 3. Results

### 3.1. Demographic and Clinical Characteristics

A total of 50 female RA patients met the eligibility criteria and were enrolled in this study receiving their first injection of ETA, ADA, CZP, or GLM (Table 1). A total of 31 patients completed the 15 months of anti-TNF-α therapy, and 19 were excluded from the analysis. Among the excluded patients with RA, anti-TNF-α therapy was discontinued for the following reasons: lack of response (five patients), loss of response (three patients), therapy intolerance (three patients), undergoing surgical procedures (four patients), and withdrawal of consent to participation in therapy (four patients). In the end, our study included 31 female patients with RA who continued the TNFαI therapy for 15 months.

During TNFαI therapy, a significant clinical improvement was noted in all RA patients. In line with the EULAR response criteria [29], 31 patients responded well after three months and this effect persisted up to month 15. The clinical parameters such as the number of tender and swollen joints, VAS, and DAS28-ESR score were significantly reduced at three, nine, and 15 months after the initiation of TNFαI therapy compared to the baseline. However, ESR and CRP levels decreased significantly only after nine and 15 months of treatment compared to the baseline (Table 3). This may indicate that in some patients—in spite of good response to the TNFαI therapy applied, as evidenced by reduction in DAS28-ESR—the treatment reduces the inflammation marker values, but they still remain above the normal range, which may entail increased cardiovascular risk in such patients. It has been demonstrated that the inflammation markers (especially ESR) are significantly correlated with the risk of cardiovascular disease (CVD) in rheumatoid arthritis [30]. Additionally, about 45% of women were over 50 years old in which reference values of ESR and CRP were higher than in women under 50 years old.

Presented in Table 3 are the variables of the demographic, clinical, and biochemical variables (except for data related to BMD and outcomes such as: serum levels of calcium, phosphorus, ALP) in rheumatoid arthritis patients during 15-month anti-TNF-α therapy that were obtained in our earlier investigations [28].

### 3.2. Bone Formation Markers—PINP and PICP

**Figure 1:**
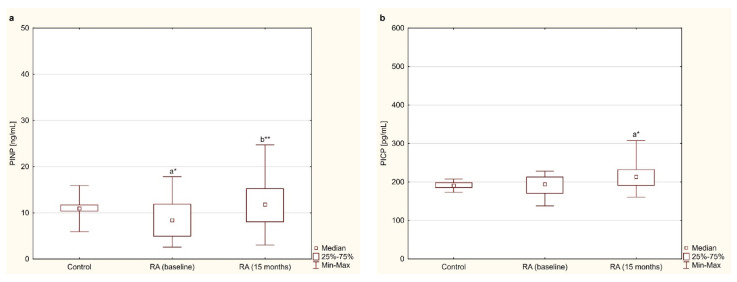
Effect of a 15-month TNFαI therapy on markers of bone formation: PINP (**a**) and PICP (**b**) in women with rheumatoid arthritis, and in healthy women. ^a^* *p* < 0.01 compared to the control; ^b^** *p* < 0.001 compared to the baseline. PICP, C-terminal propeptides of type I procollagen; PINP, N-terminal propeptides of type I procollagen; RA, rheumatoid arthritis; TNFαI, tumor necrosis factor α inhibitors.

The serum levels of the bone formation marker (i.e., PINP) were lower in women with RA before anti-TNF-α therapy than in healthy women (*p* < 0.01; Figure 1a). In addition, a significant increase in PINP levels in women with RA was found after 15 months of anti-TNF-α therapy compared to the baseline values (*p* < 0.001; Figure 1a). Regarding the second marker of bone formation (i.e., PICP), it was shown that its levels in blood serum did not differ in women with RA before anti-TNF-α therapy compared to the values in healthy subjects (*p* = 0.488; Figure 1b). Fifteen months after starting TNFαI therapy, a statistically significant increase in PICP levels was observed in RA patients compared to the healthy controls (*p* < 0.01; Figure 1b). However, PICP levels did not differ after 15 months of anti-TNF-α treatment compared to the baseline (*p* = 0.295; Figure 1b).

### 3.3. Bone Resorption Markers—CTX-I and NTX-I

**Figure 2:**
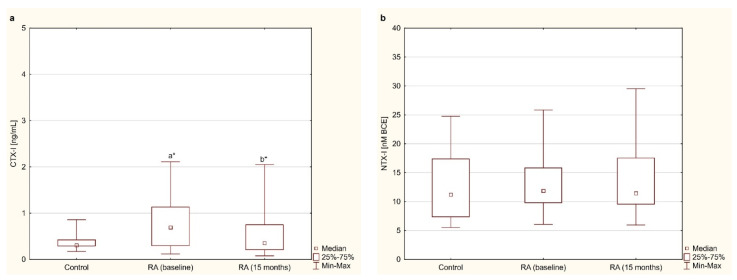
Effect of a 15-month TNFαI therapy on markers of bone resorption: CTX-I (**a**) and NTX-I (**b**) in women with rheumatoid arthritis, and in healthy women. ^a^* *p* < 0.01 compared to the control; ^b^* *p* < 0.01 compared to the baseline. CTX-I, C-terminal cross-linking telopeptides of type I collagen; NTX, N-terminal cross-linking telopeptides of type I collagen; RA, rheumatoid arthritis; TNFαI, tumor necrosis factor α inhibitors.

The serum concentrations of CTX-I and NTX-I in women with RA before and after 15 months of anti-TNF-α therapy and in healthy subjects are presented in Figure 2a,b. Before anti-TNF-α treatment, serum levels of CTX-I were significantly higher in women with RA than in healthy subjects (*p* < 0.01; Figure 2a). In addition, in women with RA, CTX-I levels significantly decreased after 15 months of anti-TNF-α therapy compared to the baseline (*p* < 0.01; Figure 2a). Regarding the second marker of bone resorption (i.e., NTX-I), it was shown that its levels in blood serum did not differ in women with RA before and after 15 months of anti-TNF-α therapy compared to the values in healthy subjects (*p* = 0.385 and *p* = 0.403, respectively; Figure 2b). Moreover, the serum levels of NTX-I in RA patients did not show any differences during the 15 months of anti-TNF-α treatment (*p* = 0.595; Figure 2b).

### 3.4. Osteoclastogenesis Markers—sRANKL and OPG

**Figure 3:**
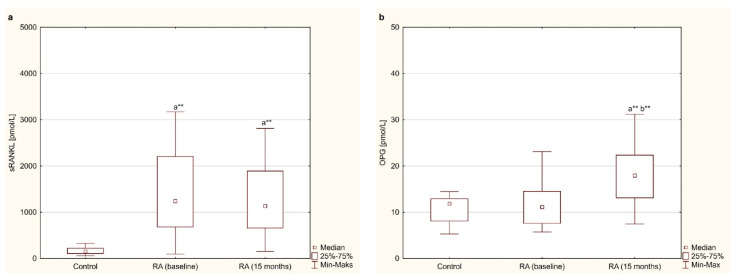
Effect of a 15-month TNFαI therapy on markers of osteoclastogenesis: sRANKL (**a**) and OPG (**b**) in women with rheumatoid arthritis, and in healthy women. ^a^** *p* < 0.001 compared to the control, ^b^** *p* < 0.001 compared to the baseline. OPG, osteoprotegerin; RA, rheumatoid arthritis; sRANKL, total soluble receptor activator of nuclear factor κB ligand; TNFαI, tumor necrosis factor α inhibitors.

Among the markers of osteoclastogenesis, serum levels of sRANKL were significantly higher in women with RA before and after 15 months of anti-TNF-α therapy than in healthy subjects (*p* < 0.001 for both; Figure 3a). Moreover, with regard to the RANKL pathway, serum levels of sRANKL in RA patients did not show any differences during 15 months of anti-TNF-α therapy (*p* = 0.281; Figure 3a). On the other hand, in the case of OPG, it was shown that the serum levels of OPG in women with RA before treatment with TNF-α inhibitors did not differ from those in healthy subjects (*p* = 0.343; Figure 3b). However, in contrast to sRANKL, 15-month of anti-TNF-α therapy resulted in a significant increase in OPG concentration compared to the baseline level (*p* < 0.001; Figure 3b). Moreover, serum OPG were still significantly higher in RA patients after 15 months of treatment than in healthy individuals (*p* < 0.001; Figure 3b). 

When calculating bone formation/resorption ratios (PINP/CTX-I and sRANKL/OPG), which better reflect bone turnover in RA, the PINP/CTX-I ratio increased significantly after 15 months of treatment compared to the baseline (*p* < 0.001; Table 4), reaching the same value as in healthy people (*p* = 0.469; Table 4). On the other hand, the sRANKL/OPG ratio was significantly higher in women with RA before and after 15 months of anti-TNF-α therapy compared to healthy subjects (*p* < 0.001 for both; Table 4). The applied treatment resulted in a significant decrease in the sRANKL/OPG ratio in women with RA (*p* < 0.001; Table 4), but was still higher than in healthy women (*p* < 0.001; Table 4).

### 3.5. Analysis of the Relationship between Bone Turnover Markers, Osteoclastogenesis Markers as Well as Clinical and Laboratory Indicators of Disease Activity

The analysis of the relationship between selected biomarkers of both bone turnover (PINP, CTX-I and PINP/CTX-I ratio) and osteoclastogenesis (sRANKL, OPG, sRANKL/OPG ratio) as well as clinical (DAS 28-ESR) and laboratory (ESR, CRP) indicators of disease activity in RA patients at the beginning and after 15 months of anti-TNF-α therapy are presented in Table 5. However, no significant correlations were found between the biomarkers of bone turnover and osteoclastogenesis with any of the parameters assessed.

### 3.6. Effect of Anti-TNF-α Treatment on Bone Mineral Density

In the RA patients, anti-TNF-α treatment halted further generalized bone loss over 15 months. During the 15-month anti-TNF-α therapy, no significant difference was found in the BMD of vertebrae (L2–L4) and femoral neck (*p* = 0.290 and *p* = 0.513, respectively; Table 3). Moreover, the rates of T-score and Z-score for L2–L4 vertebral (*p* = 0.838 and *p* = 0.510, respectively; Table 3) as well as femur neck (*p* = 0.280 and *p* = 0.09, respectively; Table 3) did not differ in women with RA before anti-TNF-α treatment compared to the values in 15 months of these therapy.

### 3.7. Bone Metabolism Markers and PINP/CTX-I, sRANKL/OPG Ratios Depending on the Type of TNF-α Inhibitor Used

In this study, we assessed whether the type of TNF-α inhibitor used had an effect on the levels of bone turnover markers. We compared changes in serum levels of bone turnover markers (PINP, CTX-I, PINP/CTX-I ratio) and osteoclast regulators (sRANKL, OPG, sRANKL/OPG ratio) in females with RA who completed a 15-month anti-TNF-α therapy with ETA or ADA. The effect of certolizumab pegol was not assessed because in this study only two patients were treated with this drug and completed 15 months of treatment with a TNF-α inhibitor (Table 3).

**Figure 4:**
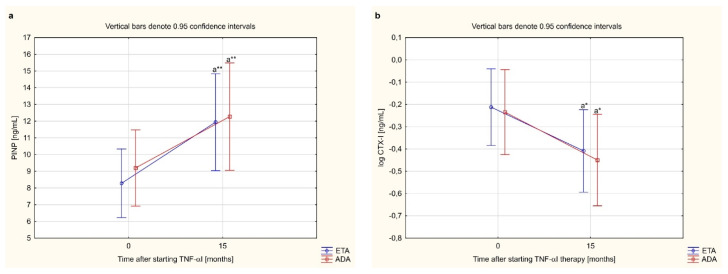

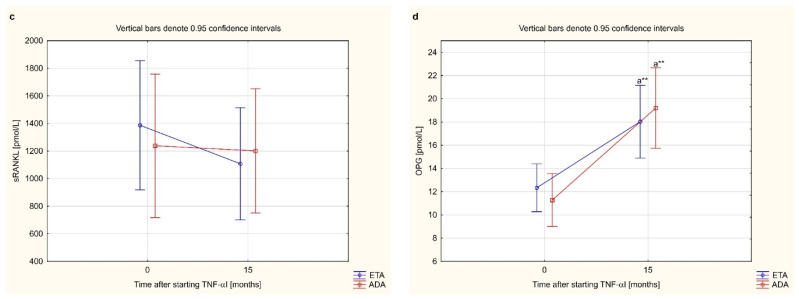
The effect of type TNF-α inhibitor used on serum levels of bone turnover markers, i.e. PINP (**a**), CTX-I (**b**) and osteoclastogenesis markers: sRANKL (**c**) and OPG (**d**) in female rheumatoid arthritis patients during 15-month therapy. ^a^* *p* < 0.01 or ^a^** *p* < 0.001 compared to the baseline. ADA, adalimumab; CTX-I, C-terminal cross-linking telopeptides of type I collagen; ETA, etanercept; OPG, osteoprotegerin; PINP, N-terminal propeptides of type I procollagen; RA, rheumatoid arthritis; sRANKL, total soluble receptor activator of nuclear factor κB ligand; TNFαI, tumor necrosis factor α inhibitors.

**Figure 5:**
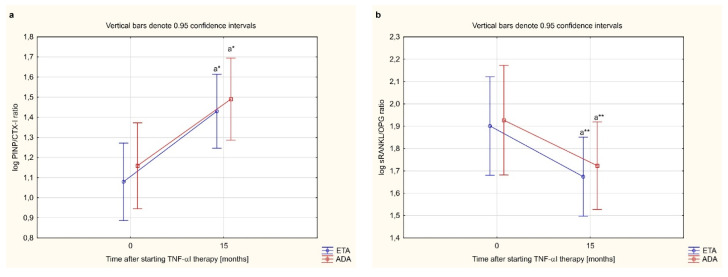
The effect of type TNF-α inhibitor used on PINP/CTX-I (**a**) and sRANKL/OPG ratios (**b**) in female rheumatoid arthritis patients during 15-month therapy. ^a^* *p* < 0.01 or ^a^** *p* < 0.001 compared to the baseline. ADA, adalimumab; CTX-I, C-terminal cross-linking telopeptides of type I collagen; ETA, etanercept; OPG, osteoprotegerin; PINP, N-terminal propeptides of type I procollagen; RA, rheumatoid arthritis; sRANKL, total soluble receptor activator of nuclear factor κB ligand; TNFαΙ, tumor necrosis factor-α inhibitors.

Administration of either ETA or ADA led to a significant increase in serum PINP and OPG levels, and decrease in serum CTX-I levels in women with RA after 15 months of therapy (*p* < 0.001, *p* < 0.001, and *p* < 0.01, respectively; Figure 4a,b,d). Moreover, there were no significantly changes in sRANKL concentration during 15 months anti-TNF-α therapy (*p* = 0.326, Figure 4c). We also demonstrated that therapy with either ETA or ADA significantly increased the PINP/CTX-I ratio (*p* < 0.01; Figure 5a), and decreased the sRANKL/OPG (*p* < 0.001; Figure 5b). There were no significant changes in the serum levels of the assessed bone turnover markers, PINP, CTX-I and PINP/CTX-I ratio (*p* = 0.713, *p* = 0.891 and *p* = 0.576, respectively; Figure 4a,b and Figure 5a) and osteoclast regulators, sRANKL, OPG, and sRANKL/OPG ratio depending on the type of TNF-α inhibitor used (*p* = 0.450, *p* = 0.345, and *p* = 0.841, respectively; Figure 4c,d and Figure 5b).

## 4. Discussion

Bone erosion is one of the radiographic hallmarks of RA. Periarticular bone loss in patients with RA results from excessive local bone resorption and inadequate bone repair [3,5,8,10]. The evaluation of bone turnover markers (BTMs) in body fluids (i.e., synovial fluid, blood, and urine) provides information on the dynamics of bone matrix turnover and reflects the disease activity in bone tissue [31,32]. Approximately 90% of the organic matrix of bone tissue is type I collagen [33]. During type I collagen synthesis, two extension peptides from both ends of the procollagen molecule, C-terminal propeptide of type I procollagen and N-terminal propeptide of type I procollagen, are enzymatically removed from the structure and released into the circulation. Although type I collagen propeptides may also arise from other tissues such as skin, dentin, tendon, and cartilage, these tissues exhibit a slower turnover than bone and make a very small contribution to the pool of circulating propeptide. Thus, PICP and PINP serum concentrations depend mainly on intrinsic bone formation. Meanwhile, C-terminal and N-terminal crosslinking telopeptides of type I collagen are formed during bone collagen breakdown with the enzyme cathepsin K, and their serum levels indicate bone resorption and osteoclast activation [31,32,34]. In the present study, we evaluated the usefulness of BTMs in predicting and monitoring bone metabolism changes during 15-months of anti-TNF-α therapy in female patients with RA. We demonstrated that the effective 15-month anti-inflammatory treatment with TNFαI was associated with improvement in bone metabolism, assessed through serum levels of matrix products released during bone formation (PICP, PINP) and bone resorption (CTX-I, NTX-I). Indeed, higher levels of PINP and lower levels of CTX-I were noted under anti-TNF-α treatment. Thus, circulating PINP and CTX-I turned out to be useful for monitoring bone turnover during TNFαI therapy. Similar results regarding persistent decrease in bone resorption markers and improvement in bone formation markers have been reported in other studies evaluating the effect of therapy with biological agents on bone turnover [21,22,23,35,36]. Vis et al. [22] described a decrease in the bone resorption marker (i.e., cross-linked C-terminal telopeptides of type I collagen (ICTP)), and an increase in the bone formation markers (i.e., PINP and osteocalcin (OC)) after six weeks of treatment with infliximab in RA patients. Chopin et al. [21] also observed this positive effect in a study with RA patients treated with infliximab for one year. There was an initial decrease in bone resorption marker (i.e., CTX-I) at week 6 and 22, which returned to pretreatment levels at week 54 [21].

In our study, the PINP/CTX-I ratio, which better reflects the bone turnover in RA, was also calculated. We noticed an increase in the PINP/CTX-I ratio in women with RA after 15 months of treatment with TNF-α inhibitors when compared to the baseline. Consistently with our results, Wheater et al. [36] and Chopin et al. [21] demonstrated that effective treatment of RA patients with biologic drugs (rituximab or infliximab) was associated with decreased serum levels of CTX-I and increased PINP/CTX-I ratio, which indicates in favor of bone formation. Moreover, Fassio et al. [37] demonstrated that the effective treatment of RA patients with certolizumab pegol in combination with MTX was associated with an increase in serum levels of PINP and a decrease in serum levels of C-terminal cross-linking telopeptides of type I collagen caused by cathepsin K, suggesting increased synthesis and decreased degradation of the bone matrix components, respectively. 

To conclude, we report that the combination of 15-month anti-TNF-α therapy with MTX provides rapid clinical response and, additionally, has a beneficial effect on bone turnover. Thus, increased serum PINP levels accompanied by significantly reduced CTX-I levels under anti-TNF-α treatment indicate a shift of bone turnover toward bone formation, which potentially represents an important mechanism for preventing future bone damage and disability in RA patients. Anti-TNF-α therapy may thus provide the ability, or at least an opportunity, to repair the damaged bone matrix due to effective disease control and inflammation suppression [13,28,38,39]. Previous studies have shown that inhibiting TNF-α in RA reduces local osteoarthritis through a reduction in synovial cell infiltration and the expression of adhesion molecules, chemokines, and cytokines, coinciding with reduced levels of acute phase reactants such as CRP and interleukin (IL)-6 [28].

We also attempted to evaluate the effect of TNFαI therapy on bone mineral density in female RA patients. In RA patients, BMD was inversely correlated with serum levels of TNF-α [13,39,40]. Thus, treatment with TNF-α inhibitors may modify BMD during this therapy. In our study, we found no significant difference in BMD of vertebrae (L2−L4) and femoral neck during 15-month anti-TNF-α therapy. It can therefore be concluded that alterations in bone marker levels (PINP and CTX-I) in response to TNFαI treatment occur earlier than changes in bone mineral density. This may indicate that anti-TNF-α treatment in female RA patients stabilized bone loss within 15-months. The anti-TNF-α therapy has also been reported to arrest bone loss in the lumbar spine and hip in longitudinal studies [18,22,41]. On the other hand, Hoff et al. [42] reported that adalimumab combined with methotrexate resulted in less hand bone loss than the use of either ADA or MTX monotherapies. Therefore, these last findings suggest that the benefits of anti-TNF-α therapy may not be limited to the control of inflammation, but also include being able to suppress the direct effect of TNF-α on osteoclast activation [41,43].

The changes in serum levels of bone resorption and bone formation markers observed in our study during therapy with TNFαI are most likely mediated by the RANKL/RANK/OPG signaling pathway. In the complex system of bone remodeling that involves the sequential phases of activation, resorption, reversal, formation, and termination, the RANKL/OPG pathway is the connecting factor between bone resorption and bone formation [12,38,44,45]. The balance between RANKL and OPG determines the degree of proliferation and activity of the osteoclasts [44,46]. The RANKL/OPG ratio, known as the regulator of osteoclastogenesis, represents an important determinant of bone resorption. In most of the cases, both RANKL upregulation and OPG downregulation lead to bone loss [45]. Moreover, as previously mentioned, various proinflammatory cytokines regulate expression of RANKL and OPG including TNF-α and IL-1 [46,47]. It was found that the high RANKL/OPG ratio in active RA is associated with increased radiographic damage in active RA [47]. Thus, the signaling and regulation of RANKL and OPG activity may play a critical role in bone loss associated with RA [44,46,47].

We have found that circulating levels of sRANKL were much higher in women with RA before anti-TNF-α treatment than in healthy subjects of the same age. Our finding of increased sRANKL in RA patients was similar to that revealed in many other studies [46,48,49]. Previously, Ziołkowska et al. [48], and Xu et al. [46], also reported elevated sRANKL levels in RA patients when compared to controls. High levels of RANKL were found in synovial fluid of patients with RA [50] in previous studies. Elevated RANKL in RA is probably related to the effect of activity of inflammatory cytokines, such as TNF-α, IL-1, IL-6, and IL-17, with regard to synovial fibroblasts [51]. In addition, patients with early RA who had high levels of sRANKL and low levels of OPG in synovial fluid experienced more rapid progression of the disease towards destruction of joints and bones [12].

We did not find any significant change in sRANKL in female RA patients undergoing treatment with TNFαI. Similar results were reported after anti-TNF-α treatment [52] and by study on treatment with methotrexate only [51]. However, some studies have found normalization or reduction of elevated sRANKL levels after treatment with both TNFαI [48] and DMARDs regimens [53]. These discrepancies can result from methodological differences, especially with regard to the specificity of antibodies used in enzyme immunoassays, capable of recognizing soluble RANKL present in the serum as a free or OPG-bound molecule. Furthermore, since sRANKL may originate from sources other than bone, it is reasonable that the circulating sRANKL levels may not entirely reflect the bone microenvironment in RA patients following the anti-TNF-α treatment [54,55]. In addition, the lack of decrease in sRANKL levels in women undergoing TNFαI therapy that we found during our research may be related to patient age. In the studies of Ziołkowska et al. [48], it was demonstrated that the reduction in sRANKL levels caused by anti-TNF-α treatment was more significant among older RA patients rather than young ones.

As has been mentioned earlier, the balance between bone breakdown and formation is modulated to a large extent by the OPG [56]. The latter one is secreted by osteoblasts, as well as by other cell types, including peripheral blood lymphocytes, endothelial cells (ECs), and vascular smooth muscle cells (SMCs) [54,56,57]. We found that circulating levels of OPG, a member of tumor necrosis factor receptor superfamily 11B (TNFRSF11B) in women with RA before treatment with TNFαI did not differ from those in healthy subjects, while the applied treatment resulted in a significant increase in OPG in women with RA. Our findings differ from those of Ziołkowska et al. [48] and Xu et al. [46], who demonstrated that the levels of OPG were higher in serum of RA patients when compared to healthy controls. On the other hand, Fadda et al. [49] found a decrease in serum OPG and an increase in serum RANKL in RA patients. Such major discrepancies in OPG levels found by different authors may be related, among others, to the impact of various factors on the levels of this marker. Ziołkowska et al. [48] underlined the impact of age on OPG levels. These authors found a significant increase in OPG levels only in RA patients below the age of 48 [48]. Meanwhile, the increased OPG levels in women after 15-month TNFαI therapy that we revealed in our studies may constitute a compensation mechanism limiting bone erosion, as in the case of increased bone turnover disorders, such as osteoporosis [58]. Moreover, previous studies have reported that balanced bone turnover, protects against the progression of vascular calcification and the occurrence of cardiovascular events [59]. It is well known that inflammation which occurs in the RA is one of the main factors responsible for the premature, rapid development of atherosclerosis, as well as for increased morbidity and mortality due to cardiovascular diseases [60,61]. High amounts of OPG can be found in the arterial wall, and the concentration found in human aorta extracts has been at least as high as in the bone extracts and even 1000 times higher in comparison to the concentration in plasma [62]. This may suggest that arterial SMCs might be major contributors to the circulating pool of OPG in RA patients. However, little is known about the functions of OPG in the arterial wall. Experimental data and clinical observations appear rather conflicting [62]. Although several studies have shown that high levels of circulating OPG are associated with cardiovascular mortality in elderly women and cardiovascular disease in the general population, vessel wall-derived OPG was also found to protect from atherosclerosis and vascular calcification in apolipoprotein E-deficient (ApoE-/-) mice [54,57,63,64,65,66]. Thus, these studies point to an active role for OPG in the maintenance of cardiovascular homeostasis. However, more evidence is needed to evaluate the predictive and diagnostic value of serum OPG levels for clinical use as a potential marker of CVD risk. To summarize, we can suppose that increased circulating levels of OPG following anti-TNF-α therapy may represent an insufficient compensatory self-defensive mechanism aimed at preventing further vascular damage in patients with RA. This is consistent with the unchanged serum calcium levels found in our study in women with RA after 15 months of anti-TNF-α treatment.

Previous studies have indicated that the RANKL/OPG ratio has a better prognostic value in assessing bone metabolism or the effectiveness of the therapy applied than the results of quantitative analysis of each of these molecules carried out in separation [12,34,50,67]. As has been demonstrated, the sRANKL/OPG ratio may be a determinant of activation in bone resorption: a high RANKL/OPG ratio is a better indicator of osteoclastogenesis and, therefore, of bone erosion in RA [52]. Moreover, it better reflects the combined effect of the two opposing osteoimmunological mediators (sRANKL and OPG). Several clinical studies have shown that the sRANKL/OPG ratio in serum or synovial fluid may predict the progression of joint and bone destruction in RA patients [16,52]. Furthermore, a recent study demonstrated that the RANKL/OPG ratio independently predicted annual radiological damage over 11 years in early RA [16].

Our study has shown that the sRANKL/OPG ratio was significantly higher in women with RA before and after 15 months of anti-TNF-α therapy when compared to healthy subjects. However, the treatment applied resulted in a significant decrease in the sRANKL/OPG ratio in women with RA. Results similar to ours were found in previous studies [48,50]. Catrina et al. [50] demonstrated a decreased expression of RANKL/OPG in synovial tissue caused by anti-TNF-α therapy through upregulation of OPG. The research so far has shown that systemic TNF-α contributes directly to increased presence of osteoclast precursors in mice, which can be reversed by applying anti-TNF antibody-based drugs [68,69]. Moreover, anti-TNF-α treatment in psoriatic arthritis patients reduced the number of peripheral osteoclast precursors [50,70]. It is plausible, therefore, that the bone-protective effect of TNFαI that we found in our study and that can be seen in women with RA receiving treatment with either ETA or ADA is, at least partially, mediated through the RANKL/OPG pathway.

Regarding the effects of the type of TNF-α inhibitor employed in our study (i.e., etanercept or adalimumab) on the levels of bone turnover markers (PINP, CTX-I) and osteoclast regulators (sRANKL, OPG) in female RA patients, our results have not demonstrated any superiority of ETA therapy in terms of preventing bone damage when compared to ADA. However, a definitive confirmation of our results require further studies in light of the relatively small number of patients in the studied groups.

Our study has several potential limitations. First, the group of women with RA was relatively small but carefully selected according to the Polish National Health Fund Therapeutic Programs that employ TNF-blockers (i.e., B.33: “Treatment of aggressive forms of rheumatoid arthritis (RA) and juvenile idiopathic arthritis (JIA)” (03.0000.333.02) or B.45: “Treatment of an aggressive form of rheumatoid arthritis (03.0000.345.02)”). Moreover, in our study, 19 (38%) female patients discontinued TNFαI treatment due to the following reasons: no response in two patients, loss of response in three patients, intolerance in three patients, surgical procedures in four patients, and withdrawal of consent for participation in the therapy by four patients. The relatively small number limits the potential for detecting smaller changes in variables and although 31 patients responded well to TNFαI treatment, the group was heterogeneous, consisting of pre- and postmenopausal women. Second, PICP as a bone formation marker and NTX-I as a resorption marker have a limited role in the evaluation of bone remodeling for the purposes of assessing the effectiveness of TNFαI therapy. PICP is cleared by the mannose receptor, which is also regulated by thyroid hormone and growth hormone. NTX-I measurements are altered in the cases of liver and renal failure. Another limitation of this study is the fact that both RANKL and OPG levels can be assayed in serum and reflect the production coming from several tissues. Therefore, the circulating levels of osteoclast regulatory proteins may not entirely reflect the bone microenvironment.

## 5. Conclusions

In conclusion, we report that a 15-month TNFαI therapy combined with MTX not only leads to clinical response, but also provides a beneficial effect on bone turnover. Changes in bone turnover marker levels occur independently of changes in BMD, which may suggest an important role for PINP and CTX-I as short-term tools to monitor bone turnover in RA patients treated with TNFαI. Our study has also demonstrated that TNFαI therapy modulates the RANKL/OPG pathway, a potential mechanism that could explain the improvement of the PINP/CTX-I ratio in RA patients follow treatment with anti-TNF-α inhibitors. Furthermore, the bone metabolism changes in women with RA were independent of the type of TNFαI used.

## Figures and Tables

**Table 1:** Demographic and clinical characteristics at baseline of the 50 rheumatoid arthritis patients qualified for the treatment with TNF-α inhibitors.

Parameter	Value
Age (years)	47.52 ± 11.91
Disease duration (years)	6 (3–12)
Height (cm)	163.58 ± 6.78
Weight (kg)	65.52 ± 14.40
BMI (kg/m^2^)	24.46 ± 5.17
IgM-RF (+), *n* (%)	44 (88)
Anti-CCP (+), *n* (%)	43 (86)
ESR (mm/h)	17.0 (10.0–29.0)
CRP (mg/L)	6.37 (3.0–10.30)
SJC, *n*	7 (5–10)
TJC, *n*	12 (9–14)
VAS, (0–100 mm)	80 (70–80)
DAS 28-ESR	5.83 ± 0.49
**Anti-rheumatic drugs, *n* (%)**	
MTX (25 mg/week)	50 (100)
GC (≤7.5 mg/day)	50 (100)
FA (5 mg/day)	50 (100)
Calcium (1 g/day)	50 (100)
25-OH vitamin D (800–1000 IU/day)	50 (100)
**TNFαI therapy, *n* (%)**	
ETA (Enbrel)	24 (48)
ADA (Humira)	22 (44)
CZP (Cimzia)	2 (4)
GLM (Simponi)	2 (4)

Results are expressed as mean ± SD or median, inter-quartile (25th–75th percentile) range, or percentage (%). ADA, adalimumab; CZP, certolizumab pegol; DAS 28-ESR, disease activity score 28; ESR, erythrocyte sedimentation rate; ETA, etanercept; FA, folic acid; GC; glucocorticosteroid; GLM, golimumab; MTX, methotrexate; SD; standard deviation; SJC, swollen joint count; TJC, tender joint count; TNFαI, tumor necrosis factor α inhibitors; VAS, visual analogue scale.

**Table 2:** Demographic and clinical characteristics of the 26 healthy women.

Parameter	Value
Age (years)	46.12 ± 10.91
Height (cm)	166.64 ± 6.23
Weight (kg)	62.64 ± 8.46
BMI (kg/m^2^)	22.64 ± 2.26
ESR (mm/h)	9.00 (8.00–12.00)
RBC (10^6^/μL)	4.28 ± 0.23
Hb (g/dL)	13.08 ± 0.97
Ht (%)	38.68 ± 3.10
PLT (10^3^/μL)	263.88 ± 49.43
WBC (10^3^/μL)	8.16 ± 1.63
Glucose (mg/dL)	88.00 (86.00–95.00)
Total cholesterol (mg/dL)	181.08 ± 6.02
HDL-C (mg/dL)	59.51 ± 12.85
LDL-C (mg/dL)	95.44 ± 20.19
Triglycerides (mg/dL)	118,70 (91.90–149.70)
hsCRP (mg/L)	0.61 (0.40–2.81)
Creatinine (mg/dL)	0.86 ± 0.10
Calcium ^C^ (mmol/L)	2.27 ± 0,08
Phosphorus (mmol/L)	1.34 ± 0.25
ALP (U/L)	159.77 ± 25.18
ALT (U/L)	20.61 ± 8.35
ASP (U/L)	20.00 ± 4.55
TSH (mU/L)	2.4 (2.20–2.81)
Uric acid (mg/dL)	4.70 ± 0.70

Results are expressed as mean ± SD or median, inter-quartile (25th–75th percentile) range, or percentage (%). ALP, alkaline phosphatase; ALT, alanine transaminase; ASP, aspartate transaminase; BMI, body mass index; ^C^ albumin-corrected calcium; ESR, erythrocyte sedimentation rate; Hb, hemoglobin; hs-CRP, high sensitive-C-reactive protein; Ht, hematocrit; PLT, platelet; RBC, red blood cell; TSH, thyroid-stimulating hormone; WBC, white blood cell.

**Table 3:** The demographic, clinical, biochemical and functional variables during 15-month anti-TNF-α therapy in women with rheumatoid arthritis.

Parameter	Time after Initiation of TNFαI Therapy			
	Baseline (T_0_)	3 Months (T_1_)	9 Months (T_2_)	15 Months (T_3_)
Women with RA, *n* (%)	31 (100)			
Premenopausal females, *n* (%)	17 (54.84)			
Postmenopausal females, *n* (%)	14 (45.16)			
Age (years)	45.87 ± 12.28			
Disease duration (years)	5 (3–11)			
Growth (cm)	163.77 ± 6.63			
Weight (kg)	65.89 ± 14.60			
BMI (kg/m^2^)	24.62 ± 5.65			
IgM-RF (+), *n* (%)	28 (90.32)			
Anti-CCP (+), *n* (%)	26 (83.87)			
ESR (mm/h)	17.0 (10.0–34.0)	14.0 (9.0–23.0)	13.0 (9.0–18.0) ^a^	13.0 (8.0–18.0) ^a^
CRP (mg/L)	6.3 (3.08–14.0)	4.0 (2.0–9.0)	4.0 (2.0–4.3) ^a^	4.0 (1.5–5.1) ^a^
Calcium ^C^ (mmol/L)	2.30 ± 0.11			2.31 ± 0.11
Phosphorus (mmol/L)	1.36 ± 0.20			1.37 ± 0.21
ALP (U/L)	168.5 (152.5–202)			165.5 (149.5–192)
SJC, *n*	7 (5–10)	2 (0–3) ^a,c^	0 (0–0) ^a,b^	0 (0–0) ^a,b^
TJC, *n*	12 (9–16)	4 (2–7) ^a,c^	1 (0–2) ^a,b^	0 (0–0) ^a,b,c^
VAS, (0–100 mm)	80 (80–80)	40 (30–50) ^a,c^	20 (10–30) ^a,b^	15 (5–20) ^a,b^
DAS 28-ESR	5.78 (5.51–6.24)	3.92 (3.08–4.42) ^a,c^	2.75 (2.24–3.13) ^a,b^	2.19 (1.75–2.51) ^a,b,c^
Disease activity, *n* (%)				
High (>5.1)	31 (100)	2 (6.45)	0 (0)	0 (0)
Moderate (>3.2 and ≤5.1)	0 (0)	20 (64.52)	3 (9.68)	0 (0)
Low (≤3.2 and >2.6)	0 (0)	4 (12.91)	14 (45.16)	5 (16.13)
Remission (≤2.6)	0 (0)	5 (16.13)	14 (45.16)	26 (83.87)
Lumbar L2-L4 BMD (g/cm^3^)	0.89 (0.73–1.00)			0.92 (0.79–1.03)
Lumbar L2-L4 T-score	−2.05 (−2.93–0.32)			−1.70 (−2.75–-0.65)
Lumbar L2-L4 Z-score	−1.43 (−2.38–0.23)			−1.15 (−2.00–0.15)
Neck femur BMD (g/cm^3^)	0.83 (0.69–0.78)			0.85 (0.77–0.85)
Neck femur T-score	−0.30 (−1.30–0.40)			−0.30 (−1.8–0.50)
Neck femur Z-score	−0.10 (−0.90–0.10)			0.00 (−0.70–0.10)
**Patients which responded to TNFαI therapy, *n* (%)**				
ETA (Enbrel)	16 (51.62)			
ADA (Humira)	13 (41.93)			
CZP (Cimzia)	2 (6.45)			

Results are expressed as mean ± SD or median, inter-quartile (25th–75th percentile) range, or percentage (%). Differences noted for all variables (except for data related to BMD and outcomes such as serum levels of calcium, phosphorus, ALP) considered significant at *p* < 0.0083 by applying Bonferroni correction. ^a^ significant differences compared to T_0_; ^b^ significant differences compared to T_1_; ^c^ significant differences compared to T_2_. ADA, adalimumab; ALP, alkaline phosphatase; BMD, bone mineral density; ^C^ albumin-corrected calcium; CZP, certolizumab pegol; DAS 28-ESR, disease activity score 28; ETA, etanercept; ESR, erythrocyte sedimentation rate; SJC, swollen joint count; TJC, tender joint count; TNFαI, tumor necrosis factor α inhibitors; VAS, visual analogue scale.

**Table 4:** The ratios of PINP to CTX-I and sRANKL to OPG in women with rheumatoid arthritis at baseline and after 15 months of anti-TNF-α therapy, and in healthy women.

Parameter	Healthy Subjects	RA Patients (*n* = 31)		*p*		
		Time after Initiation of TNFαI Therapy				
	A	Baseline (T_0_) B	15 Months (T_3_) C	A vs. B	A vs. C	B vs. C
PINP/CTX-I ratio	33.79 (25.15–48.31)	12.30 (6.69–20.20)	30.74 (14.51–50.06)	<0.001	NS	<0.001
sRANKL/OPG ratio	14.77 (10.20–23.97)	107.21 (39.20–190.12)	52.87 (34.25–94.28)	<0.001	<0.001	<0.001

Results are expressed as median, inter-quartile (25th–75th percentile) range. CTX-I, C-terminal cross-linking telopeptides of type I collagen; NS, not significant; OPG, osteoprotegerin; PINP, N-terminal propeptides of type I procollagen; RA, rheumatoid arthritis; sRANKL, total soluble receptor activator of nuclear factor κB ligand; TNFαΙ, tumor necrosis factor α inhibitors.

**Table 5:** The relationship between markers of bone turnover and osteoclastogenesis markers as well as PINP/CTX-I, sRANKL/OPG ratios, and clinical and laboratory indicators of disease activity in women with rheumatoid arthritis at the baseline and after 15 months of anti-TNF-α therapy.

Parameter	RA Patients (*n* = 31) Time after Initiation of TNFαI Therapy					
	**Baseline (T_0_)**					
	PINP	CTX-I	PINP/CTX-I	sRANKL	OPG	sRANKL/OPG
CRP	−0.167 ^NS^	−0.216 ^NS^	0.03 ^NS^	0.238 ^NS^	−0.05 ^NS^	0.302 ^NS^
ESR	0.044 ^NS^	−0.075 ^NS^	0.213 ^NS^	0.247 ^NS^	0.233 ^NS^	0.176 ^NS^
DAS28-ESR	0.012 ^NS^	−0.216 ^NS^	0.132 ^NS^	−0.221 ^NS^	0.07 ^NS^	−0.207 ^NS^
SJC	−0.130 ^NS^	−0.223 ^NS^	0.068 ^NS^	−0.307 ^NS^	0.001 ^NS^	−0.305 ^NS^
TJC	−0.027 ^NS^	0.0245 ^NS^	−0.088 ^NS^	−0.044 ^NS^	−0.236 ^NS^	−0.320 ^NS^
VAS	0.105 ^NS^	0.146 ^NS^	−0.097 ^NS^	−0.047 ^NS^	0.101 ^NS^	−0.160 ^NS^
	**15 Months (T_3_)**					
CRP	−0.08 ^NS^	0.319 ^NS^	−0.333 ^NS^	−0.03 ^NS^	−0.128 ^NS^	0.059 ^NS^
ESR	0.202 ^NS^	0.067 ^NS^	0.145 ^NS^	0.169 ^NS^	0.285 ^NS^	0.091 ^NS^
DAS28-ESR	0.185 ^NS^	0.112 ^NS^	0.054 ^NS^	0.193 ^NS^	0.255 ^NS^	0.077 ^NS^
SJC	0.353 ^NS^	0.085 ^NS^	0.097 ^NS^	0.000 ^NS^	−0.036 ^NS^	0.012 ^NS^
TJC	−0.028 ^NS^	−0.028 ^NS^	0.0176 ^NS^	0.097 ^NS^	−0.214 ^NS^	0.051 ^NS^
VAS	0.082 ^NS^	0.040 ^NS^	−0.047 ^NS^	0.071 ^NS^	0.322 ^NS^	−0.091 ^NS^

Results are expressed as r (correlation coefficient) according to Spearman rank correlation. Correlations were considered significant at: *p* < 0.0083 by applying a Bonferroni correction. DAS 28-ESR, disease activity score 28; CTX-I, C-terminal cross-linking telopeptides of type I collagen; ESR, erythrocyte sedimentation rate; ^NS^, not significant; OPG, osteoprotegerin; PINP, N-terminal propeptides of type I procollagen; RA, rheumatoid arthritis; SJC, swollen joint count; sRANKL, total soluble receptor activator of nuclear factor κB ligand; TJC, tender joint count; TNFαΙ, tumor necrosis factor α inhibitors.

## Data Availability

Data are contained within the article.
